# Quantitative analysis of human kallikrein gene 14 expression in breast tumours indicates association with poor prognosis

**DOI:** 10.1038/sj.bjc.6600623

**Published:** 2002-11-12

**Authors:** G M Yousef, C A Borgoño, A Scorilas, R Ponzone, N Biglia, L Iskander, M-E Polymeris, R Roagna, P Sismondi, E P Diamandis

**Affiliations:** Department of Pathology and Laboratory Medicine, Mount Sinai Hospital, Toronto, Ontario, Canada; Department of Laboratory Medicine and Pathobiology, University of Toronto, Toronto, Ontario, Canada; National Center of Scientific Research ‘Demokritos’, IPC, Athens, 153 10, Greece; Academic Division of Gynecological Oncology, University of Turin, Mauriziano Umberto Hospital and Institute for Cancer Research and Treatment (IRCC) of Candiolo, Turin, Italy

**Keywords:** serine proteases, breast cancer, cancer genes, tumour markers, prognostic, predictive factors, kallikreins, *KLK14*

## Abstract

*KLK14* (formerly known as *KLK-L6*) is a recently identified member of the human kallikrein gene family. This family harbours several genes aberrantly expressed in various cancers as well as established (PSA/hK3, hK2) and potential (hK6, hK10) cancer markers. Similar to other kallikrein genes, *KLK14* was found to be regulated by steroid hormones, particularly androgens and progestins, in breast and ovarian cancer cell lines. Preliminary studies indicated that *KLK14* is differentially expressed in breast, ovarian, prostatic and testicular tumours. Given the above, we determined the prognostic significance of *KLK14* expression in breast cancer. We studied *KLK14* expression in 178 histologically confirmed epithelial breast carcinomas by quantitative reverse transcription–polymerase chain reaction and correlated with clinicopathological variables (tumour stage, grade, histotype etc.) and with outcome (disease-free survival and overall survival), monitored over a median of 76 months. *KLK14* mRNA levels ranged from 0 to 1219 arbitrary units in breast cancer tissues, with a mean±s.e. of 136±22. An optimal cutoff value of 40.5 arbitrary units was selected, to categorise tumours as *KLK14*-positive or negative. Higher concentrations of *KLK14* mRNA were more frequently found in patients with advanced stage (III) disease (*P*=0.032). No statistically significant association was found between *KLK14* and the other clinicopathological variables. *KLK14* overexpression was found to be a significant predictor of decreased disease-free survival (hazard ratio of 2.31, *P*=0.001) and overall survival (hazard ratio of 2.21, *P*=0.005). Cox multivariate analysis indicated that *KLK14* was an independent prognostic indicator of disease-free survival and overall survival. *KLK14* also has independent prognostic value in subgroups of patients with a tumour size ⩽2 cm and positive nodal, oestrogen receptor and progestin receptor status. We conclude that *KLK14* expression, as assessed by quantitative reverse transcription–polymerase chain reaction, is an independent marker of unfavourable prognosis for breast cancer.

*British Journal of Cancer* (2002) **87**, 1287–1293. doi:10.1038/sj.bjc.6600623 www.bjcancer.com

© 2002 Cancer Research UK

## 

Breast cancer is the most prevalent malignancy among women, accounting for 21% of all female cancers and ranking third overall when both sexes are considered (Parkin *et al*, 1999). Although the increased use of screening for early disease diagnosis and the widespread administration of systemic adjuvant therapies have lead to a decline in mortality rates, breast cancer is still the leading cause of death from cancer in women, causing over 39 500 deaths in the US annually (Peto *et al*, 2000; Jemal *et al*, 2002).

Given the heterogeneous nature of breast carcinomas, much attention has been focussed on the identification of tumour associated molecular markers that reveal the biological profile of each tumour and ultimately aid in determining cancer risk, diagnosis, screening, prognosis, monitoring, management and prediction of therapeutic response in breast cancer patients (Duffy, 2001). Among the multitude of markers discovered are serine proteases, which participate in many aspects of carcinogenesis, including stimulating cellular growth, angiogenesis and the degradation of the extracellular matrix (Gottesman, 1990; Duffy, 1991). These functions are in accord with clinical studies demonstrating that the aberrant expression of certain serine proteases correlates with the invasiveness and metastasis of cancer cells and predicts poor prognosis in various malignancies (Herszenyi *et al*, 1999; Kuhn *et al*, 1999).

Human kallikreins are a subset of secreted serine proteases found in a wide range of tissues and biological fluids and implicated in diverse physiological and pathological processes (Diamandis *et al*, 2000b; Yousef and Diamandis, 2001). The kallikrein genes, denoted *KLK1*–*KLK15*, are located on chromosome 19q13.4 and encode for corresponding kallikrein enzymes, hK1–hK15 (Diamandis *et al*, 2000a; Yousef *et al*, 2000b). Accumulating evidence indicates that many members of this family are differentially expressed in certain malignancies, including prostate (Rittenhouse *et al*, 1998; Magklara *et al*, 1999; Barry, 2001; Yousef *et al*, 2001c; Diamandis *et al*, 2002), testicular (Luo *et al*, 2001c), breast (Yousef *et al*, 2000a,c) and ovarian (Anisowicz *et al*, 1996; Diamandis *et al*, 2000c; Kim *et al*, 2001; Luo *et al*, 2001b; Magklara *et al*, 2001; Obiezu *et al*, 2001; Yousef *et al*, 2001a) cancers. Also, many kallikrein genes examined thus far are under steroid hormone regulation, further suggesting a role for these enzymes in endocrine-related tissues (Yousef and Diamandis, 2002). Additionally, PSA/hK3, is the best tumour marker available in clinical medicine for diagnosing and managing prostate cancer (Diamandis, 1998; Barry, 2001), hK2 is useful for certain subgroups of prostate cancer patients (Magklara *et al*, 1999), hK6, 10 and 11, have recently emerged as potential serological epithelial ovarian cancer markers (Luo *et al*, 2001a; Diamandis *et al*, 2002), and several others possess clinical utility as prognostic/predictive markers (Diamandis and Yousef, 2001).

Human kallikrein gene 14 (*KLK14*), formerly known as *KLK-L6*, is a recently identified member of the human kallikrein gene family (Yousef *et al*, 2001b). Structurally, this gene is formed of five coding exons and four intervening introns. The encoded protein, hK14, is a trypsin-like serine protease, translated as an inactive 251 amino acid preproenzyme precursor of about 27.5 kDa, of which 18 amino acids constitute the signal peptide and six amino acids the activation peptide. hK14 harbours the conserved catalytic triad characteristic of serine proteases and is highly homologous to other kallikreins, including PSA/hK3. *KLK14* has a restricted tissue expression pattern and is found in the central nervous system as well as in endocrine-related tissues such as the uterus, ovary, thyroid and testis. Additionally, *in situ* hybridisation studies demonstrated that *KLK14* is expressed by the secretory epithelial cells of benign prostate gland, prostatic intraepithelial neoplasia and malignant prostate cells (Hooper *et al*, 2001). Preliminary studies have shown that *KLK14* is down-regulated at the mRNA level in prostatic, testicular, ovarian and breast cancer tissues and in two breast cancer cell lines (Yousef *et al*, 2001b). Hormonal regulation studies in breast and ovarian cancer cell lines indicate that *KLK14* expression is controlled by the androgen receptor in response to steroid hormones, particularly androgens and progestins (our unpublished data). Based on these collective findings, we hypothesised that *KLK14* expression in malignant breast tissues may have prognostic/predictive value for patients with breast carcinomas.

## MATERIALS AND METHODS

### Study population

Tumour specimens from 178 consecutive patients undergoing surgical treatment for primary breast carcinoma at the Department of Gynecologic Oncology, University of Turin, Turin, Italy were analysed in this study. Patient age ranged from 25 to 87 years, with a median of 58 years (Table 1

**Table 1:**
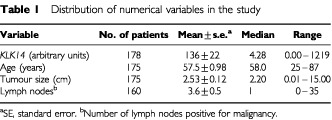
Distribution of numerical variables in the study

). Follow-up information (median follow-up period of 76 months) was available for 164 patients, among whom 60 (36%) had relapsed and 51 (31%) died. All tissue specimens were histologically confirmed and frozen in liquid nitrogen immediately after surgery.

Clinical and pathological information documented at the time of surgery included clinical stage, grade, histology and size of the tumour, number of positive axillary nodes, steroid hormone receptor status and treatment strategy (Table 2

**Table 2:**
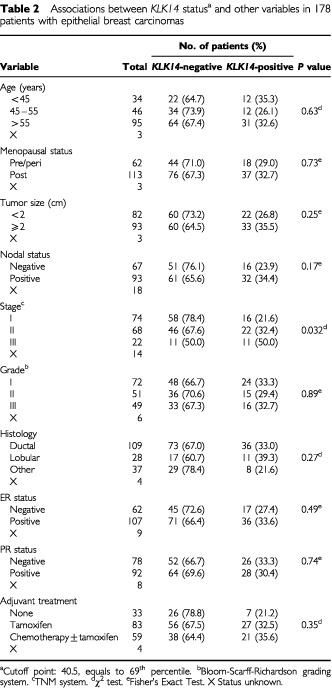
Associations between *KLK14* status^a^ and other variables in 178 patients with epithelial breast carcinomas

). Tumour sizes ranged from 0.1 to 15 cm, with a median of 2.2 cm. Out of the 178 patients, 109 (61%) had ductal carcinoma, 28 (16%) lobular carcinoma while 37 (21%) possessed other histological types. The histotype was unknown for four patients. Patients with disease of all three Stages (I–III) and tumour grades (I–III) were represented in this study, with staging determined according to the TNM classification and grading in accordance to the Bloom-Richardson grading system (Bloom and Richardson, 1957). Oestrogen and progesterone receptor status was established as described by the European Organization for Research and Treatment of Cancer (Eortc, 1980). With respect to treatment, 33 (19%) received no adjuvant treatment, 83 (47%) received tamoxifen, while 59 (33%) received chemotherapy with or without tamoxifen. This study has been approved by the Institutional Review Board of the University of Turin.

### Total RNA extraction and cDNA synthesis

Tumour tissues were minced with a scalpel, on dry ice, and transferred immediately to 2 ml polypropylene tubes. They were then homogenized and total RNA was extracted using Trizol™ reagent (Gibco–BRL) following the manufacturer's instructions. The concentration and purity of mRNA were determined spectrophotometrically. Two μg of total RNA was reverse-transcribed into first-strand cDNA using the Superscript™ pre-amplification system (Gibco–BRL). The final volume was 20 μl.

### Quantitative real-time polymerase chain reaction (PCR) and continuous monitoring of PCR products

Based on the published genomic sequence of *KLK14* (GenBank accession #AF161221), two gene-specific primers were designed (6F5: 5′-AGT GGG TCA TCA CTG CTG CT-3′ and 6R5: 5′-TCG TTT CCT CAA TCC AGC TT-3′). These primers spanned more than two exons to avoid contamination by genomic DNA.

Real-time monitoring of PCR reaction was performed using the LightCycler™ system (Roche Molecular Systems, Indianapolis, USA) and the SYBR green I dye, which binds preferentially to double-stranded DNA. Fluorescence signals are proportional to the concentration of the product and are measured at the end of each cycle and immediately displayed on a computer screen, permitting real time monitoring of the PCR reaction. The reaction is characterised at the point during cycling when amplification of PCR products is first detected, rather than the amount of PCR product accumulated after a fixed number of cycles. The higher the starting quantity of the template, the earlier a significant increase in fluorescence is observed. The threshold cycle is defined as the fractional cycle number at which fluorescence passes a fixed threshold above baseline.

### Endogenous control

For each sample, the amount of *KLK14* cDNA and of an endogenous control (β actin, a housekeeping gene) were determined using a calibration curve (see below). The amount of *KLK14* was then divided by the amount of the endogenous reference, to obtain a normalised *KLK14* value.

### Standard curve construction

The full-length mRNA sequence of the *KLK14* gene was amplified by PCR using gene-specific primers, and the PCR product was cloned into a TOPO TA cloning vector (Invitrogen, Carlsbad, CA, USA) according to the manufacturer's instructions. A plasmid containing β-actin cDNA, was similarly prepared. Plasmids were purified using a mini-prep kit (Qiagen Inc., Valencia, CA, USA). Different standard curves for actin and *KLK14* were constructed using serial dilutions of the plasmid. These standards were included in each run. The LightCycler software automatically calculates the standard curve by plotting the starting dilution of each standard sample *vs* the threshold cycle, and the sample concentrations are then calculated accordingly. Standards for both *KLK14* and actin RNAs were defined to contain an arbitrary starting concentration, since no primary preparations exist. Hence, all calculated concentrations are relative to the concentration of the selected standard. Each sample was repeated twice to ensure reproducibility.

### PCR amplification

The PCR reaction was carried out on the LightCycle™ system. For each run, a master mixture was prepared on ice, containing 1 μl of cDNA, 2 μl of LC DNA Master SYBR Green 1 mix, 50 ng of primers and 1.2 μl of 25 mM MgCl_2_. After the reaction mixture was loaded into the glass capillary tube, the cycling conditions were carried out as shown in Table 3

**Table 3:**
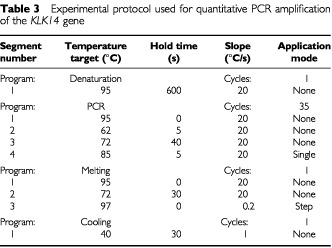
Experimental protocol used for quantitative PCR amplification of the *KLK14* gene

.

### Melting curve

For distinguishing specific from non-specific products and primer dimers, a melting curve was obtained after amplification by holding the temperature at 70°C for 30 s followed by a gradual increase in temperature to 98°C at a rate of 0.2°C per s, with the signal acquisition mode set at step. To verify the melting curve results, representative samples of the PCR products were run on 1.5% agarose gels, purified, and cloned into the pCR 2.1-TOPO vector (Invitrogen, Carlsbad, CA, USA) according to the manufacturer's instructions. The inserts were sequenced from both directions using vector-specific primers with an automated DNA sequencer.

### Statistical analysis

Patients were subdivided into groups based on different clinical or pathologic parameters (Table 2) and statistical analyses were performed using SAS software (SAS Institute, Cary, NC, USA). An optimal cutoff value was defined by χ^2^ analysis based on the ability of *KLK14* values to predict the disease-free survival (DFS) and overall survival (OS) of the study population. According to this value, tumours were categorised as *KLK14-*positive or *KLK14*-negative and associations between *KLK14* status and other qualitative variables were analysed using the χ^2^ or the Fisher's Exact test, where appropriate. The cutoff value for tumour size was 2 cm. Lymph node status was either positive (any positive number of nodes) or negative. Age was categorised into three groups: less than 45 years, 45–55 years and greater than 55 years. Survival analyses were performed by constructing Kaplan–Meier disease free survival (DFS) and overall survival (OS) curves and differences between curves were evaluated by the log-rank test (Mantel, 1966), as well as by estimating the relative risks for relapse and death using the Cox proportional hazards regression model (Cox, 1972). Cox analysis was conducted at both univariate and multivariate levels. Only patients for whom the status of all variables was known were included in the multivariate regression models, which incorporated *KLK14* and all other variables for which the patients were characterised. The multivariate models were adjusted for *KLK14* expression in tumours, patient age, tumour size, stage, grade, histological type and oestrogen receptor (ER) and progestin receptor (PR) status.

## RESULTS

### Relationship between *KLK14* expression and other parameters

**Figure 1:**
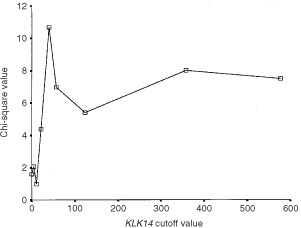
Determination of the optimal cutoff value for *KLK14* expression. For details, see text.

*KLK14* mRNA levels ranged from 0 to 1219 arbitrary units in breast cancer tissues, with a mean±s.e. of 136±22. The cutoff point (40.5 arbitrary units; 69th percentile) indicated that 55 (31%) of the 178 breast tumour tissues were positive for *KLK14* expression (Figure 1

). Table 2 depicts the distribution of *KLK14* expression, positive or negative, in breast tumour tissues in relation to other established prognostic factors such as menopausal status, tumour size, stage, grade, histological type, nodal status, steroid receptor status and adjuvant therapy. Patients with *KLK14*-positive breast tumours more frequently had advanced stage (III) disease (*P*=0.032). Significant associations between *KLK14* expression and tumour size, grade and histology, or menopausal, nodal and steroid receptor status were not observed.

### Univariate and multivariate survival analysis

The strength of association between each clinicopathological variable and DFS and OS is displayed in Table 4

**Table 4:**
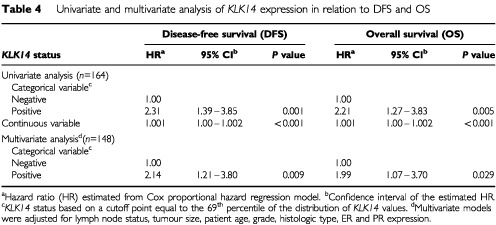
Univariate and multivariate analysis of *KLK14* expression in relation to DFS and OS

**Figure 2:**
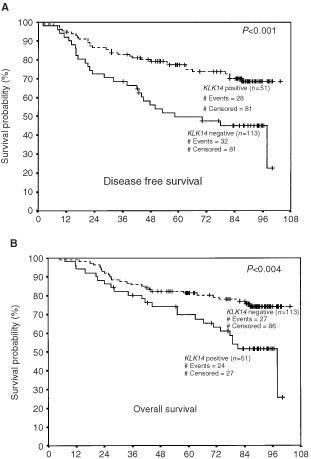
Kaplan–Meier survival curves for disease-free survival (**A**) and overall survival (**B**) in patients with *KLK14*-positive and negative breast tumours. *n*=number of samples.

. In univariate analysis, patients with *KLK14*-positive tumours had a significantly increased risk of relapse (decreased DFS) and death (decreased OS) (hazards ratios of 2.31 and 2.21; *P*=0.001 and 0.005, respectively). Importantly, when treated as a continuous variable, *KLK14* expression retained its statistically significant association with decreased DFS and OS (*P*<0.001). Further, when survival outcomes were adjusted for all other variables in the multivariate analysis (i.e. Cox proportional hazard regression model), the adverse effects of *KLK14* positivity on DFS and OS were preserved (hazards ratios of 2.14 and 1.99; *P*=0.009 and 0.029, respectively), implying that *KLK14* expression is an independent prognostic indicator. As expected, Kaplan–Meier survival curves (Figure 2

) demonstrate that patients with *KLK14-*positive tumours have shorter DFS (*P*<0.001) and OS (*P*=0.004) compared to those who are *KLK14*-negative.

### Univariate and multivariate survival analysis insubgroups of patients

We further examined the associations between *KLK14* expression levels and survival outcomes in subgroups of patients stratified by tumour size, nodal, OR and PR status (Table 5

**Table 5:**
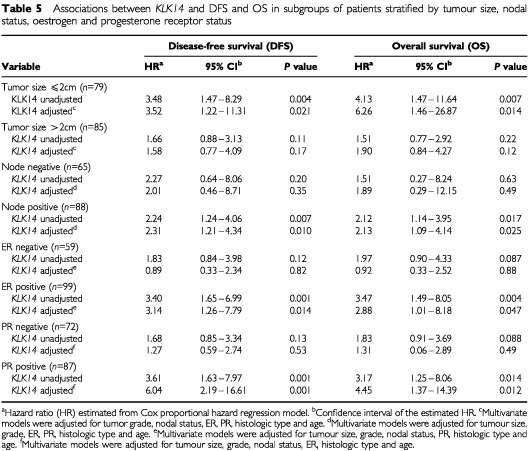
Associations between *KLK14* and DFS and OS in subgroups of patients stratified by tumour size, nodal status, oestrogen and progesterone receptor status

). *KLK14* expression significantly impacted survival in subgroups of patients with a tumour size ⩽2 cm and positive nodal, OR and PR status. Univariate analysis revealed that *KLK14-*positive patients with a tumour size ⩽2 cm were about three times more likely to suffer disease progression and four times more likely to die than *KLK14*-negative patients (*P*=0.004 and 0.007, respectively). These survival differences remained significant after the data were subjected to multivariate analysis (*P*=0.021 and 0.014, respectively). Among patients with positive nodal status, high *KLK14* expression was associated with approximately two-fold greater risk of relapse and death in both univariate (*P*=0.007 and 0.017, respectively) and multivariate analyses (*P*=0.010 and 0.025, respectively). Similarly, there was a tendency for an increased risk of disease progression and death in both OR and PR positive patients with *KLK14*-positive tumours. In univariate analysis, hazard ratios derived from the Cox regression model in relation to DFS and OS were 3.40 (*P*=0.001) and 3.47 (*P*=0.004) respectively, for ER positive and 3.61 (*P*=0.001) and 3.17 (*P*=0.014) for PR positive patients. Under multivariate analysis, *KLK14* retained its independent prognostic value in these subgroups of patients.

## DISCUSSION

The optimal management of women with breast cancer involves a multidisciplinary approach, including the use of biological markers. Ultimately, the goal is to select the best marker or panel of markers that are most informative in terms of their ability to predict relapse, disease progression, survival and response to therapy. Traditional prognostic/predictive factors in breast cancer include tumour size, grade, nodal status, hormone receptor status, vascular invasion and age (Denley *et al*, 2001). However, only hormone receptor status has predictive value in selecting patients who are likely to respond to therapy, and is the only marker recommended for routine use by the American Society of Clinical Oncology (Smith *et al*, 2001) and the College of American Pathologists Consensus Statement (Fitzgibbons *et al*, 2000).

Many other potential biological markers have been identified as prognostic factors including p53, c-*myc*, BCL-2, HER-2, vascular endothelial growth factor (VEGF), urokinase plasminogen activator (uPA), CA 15-3, BR 27.29 and cathepsin B and D (Maguire *et al*, 1998; Duffy *et al*, 1999; Scorilas *et al*, 1999; Hamilton and Piccart, 2000; Bundred, 2001). Some markers are also predictive. For instance, expression of HER-2 has value in determining response to treatment and in selecting patients for Herceptin therapy (Ross and Fletcher, 1998). The multitude of new candidate biomarkers will likely lead to insights into the cellular changes that correlate with, or determine the different biological properties and diverse behaviour of individual breast tumours. Furthermore, stratifying patients based on the presence or absence of such markers may help to tailor different therapeutic strategies to meet individual needs (Clark *et al*, 1994).

In this paper, we identify *KLK14* as a new independent marker of unfavourable prognosis in breast cancer. Patients with *KLK14*-positive breast tumours were more likely to have advanced stage (III) disease. When assessing *KLK14* expression in terms of predicting survival outcomes, we found an increased risk of relapse and death for patients with *KLK14*-positive tumours. This was also observed in subgroups of patients with a tumour size ⩽2 cm, positive nodal, OR and PR status. Hence, *KLK14* expression may aid in predicting relapse, disease progression and/or survival in breast cancer patients.

Interestingly, the results obtained for *KLK14* in this study are comparable to those obtained for *KLK3*, *KLK6* and *KLK15* in breast cancer (Yu *et al*, 1995; Anisowicz *et al*, 1996) and *KLK4*, *KLK5* and *KLK10* in ovarian cancer (Kim *et al*, 2001; Luo *et al*, 2001b; Obiezu *et al*, 2001), in that high expression of these kallikrein genes also correlated with patient prognosis. Similar to *KLK14*, these genes are also under steroid hormone regulation (Yousef and Diamandis, 2001). These observations allow us to speculate that multiple kallikreins may participate in a common enzymatic pathway that plays a role in the normal physiology of the breast. This pathway may be deregulated in breast carcinogenesis. As is the case with many other serine proteases, certain kallikreins may degrade the extracellular matrix promoting tumour invasiveness and metastasis.

It was recently realised that *KLK14* is under steroid hormone regulation, particularly androgens and progestins, and that these effects are mediated through the androgen receptor (our unpublished data). It is also known that breast cancer is a hormone-dependent malignancy (Russo and Russo, 1998) and that androgen receptors are present in 70–90% of primary breast tumours (Soreide *et al*, 1992), and 75% of breast cancer metastases (Lea *et al*, 1989). Thus, it is likely that the androgen receptor, acting as a ligand-activated transcription factor, upregulates *KLK14* gene expression during breast carcinogenesis.

The role of androgens in the aetiology of breast cancer is ill-defined. *In vitro*, they both stimulate (MCF-7, MDA-453) or inhibit (T-47D, ZR-75-1, MFM-223) the growth of AR-positive breast cancer cell lines (Hackenberg *et al*, 1991; Birrell *et al*, 1995). Animal studies have demonstrated that androgens shorten the latency period, enhance tumour size and increase the incidence of breast tumours, by promoting rather than initiating carcinogenesis in rodents (Liao *et al*, 1998; Xie *et al*, 1999). These effects may be mediated by androgen-regulated genes that directly function in cell growth regulation and through interaction of the AR with other transcription regulators, allowing for cross-talk with other growth pathways (Brys, 2000). Thus, the identification of androgen regulated genes, such as *KLK14*, may help to define new targets for breast cancer treatment. Such new treatments may be particularly important in metastatic disease, where the AR is often the sole steroid receptor expressed.

In summary, we quantified *KLK14* expression in breast tumours and found that high *KLK14* expression is associated with decreased DFS and OS in both univariate and multivariate analysis. Additional basic and clinical studies are required to delineate the activity of *KLK14* in both the normal and malignant breast and to further define the clinical value of this biomarker.

## References

[bib1] AnisowiczASotiropoulouGStenmanGMokSCSagerR1996 A novel protease homolog differentially expressed in breast and ovarian cancer Mol Med 2 624636 8898378 PMC2230195

[bib2] BarryMJ2001 Clinical practice. Prostate-specific-antigen testing for early diagnosis of prostate cancer N Engl J Med 344 13731377 11333995 10.1056/NEJM200105033441806

[bib3] BirrellSNBentelJMHickeyTERicciardelliCWegerMAHorsfallDJTilleyWD1995 Androgens induce divergent proliferative responses in human breast cancer cell lines J Steroid Biochem Mol Biol 52 459467 7748811 10.1016/0960-0760(95)00005-k

[bib4] BloomHJGRichardsonWW1957 Histological grading and prognosis in breast cancer Br J Cancer 11 359377 13499785 10.1038/bjc.1957.43 PMC2073885

[bib5] BrysM2000 Androgens and androgen receptor: do they play a role in breast cancer? Med Sci Monit 6 433438 11208351

[bib6] BundredNJ2001 Prognostic and predictive factors in breast cancer Cancer Treat Rev 27 137142 11417963 10.1053/ctrv.2000.0207

[bib7] ClarkGMHilsenbeckSGRavdinPMDe LaurentiisMOsborneCK1994 Prognostic factors: rationale and methods of analysis and integration Breast Cancer Res Treat 32 105112 7819579 10.1007/BF00666211

[bib8] CoxDR1972 Regression models and life tables R Stat Soc B 34 187202

[bib9] DenleyHPinderSEElstonCWLeeAHEllisIO2001 Preoperative assessment of prognostic factors in breast cancer J Clin Pathol 54 2024 11271783 10.1136/jcp.54.1.20 PMC1731277

[bib10] DiamandisEP1998 Prostate-specific antigen – its usefulness in clinical medicine Trends Endocrinol Metab 9 310316 18406295 10.1016/s1043-2760(98)00082-4

[bib11] DiamandisEPOkuiAMitsuiSLuoLYSoosaipillaiAGrassLNakamuraTHowarthDJYamaguchiN2002 Human kallikrein 11: a new biomarker of prostate and ovarian carcinoma Cancer Res 62 295300 11782391

[bib12] DiamandisEPYousefGM2001 Human tissue kallikrein gene family: a rich source of novel disease biomarkers Expert Rev Mol Diagn 1 182190 11901813 10.1586/14737159.1.2.182

[bib13] DiamandisEPYousefGMClementsJAshworthLKYoshidaSEgelrudTNelsonPSShiosakaSLittleSLiljaHStenmanUHRittenhouseHGWainH2000a New nomenclature for the human tissue kallikrein gene family Clin Chem 46 18551858 11067830

[bib14] DiamandisEPYousefGMLuoLYMagklaraAObiezuCV2000b The new human kallikrein gene family: implications in carcinogenesis Trends Endocrinol Metab 11 5460 10675891 10.1016/s1043-2760(99)00225-8

[bib15] DiamandisEPYousefGMSoosaipillaiARBuntingP2000c Human kallikrein 6 (zyme/protease M/neurosin): a new serum biomarker of ovarian carcinoma Clin Biochem 33 579583 11124344 10.1016/s0009-9120(00)00182-x

[bib16] DuffyMJ1991 The role of proteolytic enzymes in cancer invasion and metastasis Clin Exp Metast 10 145155 10.1007/BF00132746 1582084

[bib17] DuffyMJ2001 Biochemical markers in breast cancer: which ones are clinically useful? Clin Biochem 34 347352 11522269 10.1016/s0009-9120(00)00201-0

[bib18] DuffyMJMaguireTMMcDermottEWO'HigginsN1999 Urokinase plasminogen activator: a prognostic marker in multiple types of cancer J Surg Oncol 71 130135 10389872 10.1002/(sici)1096-9098(199906)71:2<130::aid-jso14>3.0.co;2-9

[bib19] EORTC1980 Revision of the standards for the assessment of hormone receptors in human breast cancer; report of the second EORTC Workshop, held on 16–17 March, 1979, in the Netherlands Cancer Institute Eur J Cancer 16 15131515 6262087 10.1016/0014-2964(80)90064-x

[bib20] FitzgibbonsPLPageDLWeaverDThorADAllredDCClarkGMRubySGO'MalleyFSimpsonJFConnollyJLHayesDFEdgeSBLichterASchnittSJ2000 Prognostic factors in breast cancer. College of American Pathologists Consensus Statement 1999 Arch Pathol Lab Med 124 966978 10888772 10.5858/2000-124-0966-PFIBC

[bib21] GottesmanM1990 The role of proteases in cancer Semin Cancer Biol 1 97160

[bib22] HackenbergRLuttchensSHofmannJKunzmannRHolzelFSchulzKD1991 Androgen sensitivity of the new human breast cancer cell line MFM-223 Cancer Res 51 57225727 1913690

[bib23] HamiltonAPiccartM2000 The contribution of molecular markers to the prediction of response in the treatment of breast cancer: a review of the literature on HER-2, p53 and BCL-2 Ann Oncol 11 647663 10942052 10.1023/a:1008390429428

[bib24] HerszenyiLPlebaniMCarraroPDe PaoliMRoveroniGCardinRTulassayZNaccaratoRFarinatiF1999 The role of cysteine and serine proteases in colorectal carcinoma Cancer 86 11351142 10506696 10.1002/(sici)1097-0142(19991001)86:7<1135::aid-cncr6>3.0.co;2-2

[bib25] HooperJDBuiLTRaeFKHarveyTJMyersSAAshworthLKClementsJA2001 Identification and characterization of klk14, a novel kallikrein serine protease gene located on human chromosome 19q13.4 and expressed in prostate and skeletal muscle Genomics 73 117122 11352573 10.1006/geno.2000.6490

[bib26] JemalAThomasAMurrayTThunM2002 Cancer statistics, 2002 CA Cancer J Clin 52 2347 11814064 10.3322/canjclin.52.1.23

[bib27] KimHScorilasAKatsarosDYousefGMMassobrioMFracchioliSPiccinnoRGordiniGDiamandisEP2001 Human kallikrein gene 5 (KLK5) expression is an indicator of poor prognosis in ovarian cancer Br J Cancer 84 643650 11237385 10.1054/bjoc.2000.1649 PMC2363783

[bib28] KuhnWSchmalfeldtBReuningUPacheLBergerUUlmKHarbeckNSpatheKDettmarPHoflerHJanickeFSchmittMGraeffH1999 Prognostic significance of urokinase (uPA) and its inhibitor PAI-1 for survival in advanced ovarian carcinoma stage FIGO IIIc Br J Cancer 79 17461751 10206287 10.1038/sj.bjc.6690278 PMC2362775

[bib29] LeaOAKvinnslandSThorsenT1989 Improved measurement of androgen receptors in human breast cancer Cancer Res 49 71627167 2582456

[bib30] LiaoDZPantazisCGHouXLiSA1998 Promotion of estrogen-induced mammary gland carcinogenesis by androgen in the male Noble rat: probable mediation by steroid receptors Carcinogenesis 19 21732180 9886575 10.1093/carcin/19.12.2173

[bib31] LuoLYBuntingPScorilasADiamandisEP2001a Human kallikrein 10: a novel tumor marker for ovarian carcinoma? Clin Chim Acta 306 111118 11282101 10.1016/s0009-8981(01)00401-6

[bib32] LuoLYKatsarosDScorilasAFracchioliSPiccinnoRRigault de la LongraisIAHowarthDJDiamandisEP2001b Prognostic value of human kallikrein 10 expression in epithelial ovarian carcinoma Clin Cancer Res 7 23722379 11489815

[bib33] LuoLYRajpert-De MeytsERJungKDiamandisEP2001c Expression of the normal epithelial cell-specific 1 (NES1; KLK10) candidate tumour suppressor gene in normal and malignant testicular tissue Br J Cancer 85 220224 11461080 10.1054/bjoc.2001.1870 PMC2364047

[bib34] MagklaraAScorilasACatalonaWJDiamandisEP1999 The combination of human glandular kallikrein and free prostate-specific antigen (PSA) enhances discrimination between prostate cancer and benign prostatic hyperplasia in patients with moderately increased total PSA Clin Chem 45 19601966 10545066

[bib35] MagklaraAScorilasAKatsarosDMassobrioMYousefGMFracchioliSDaneseSDiamandisEP2001 The human KLK8 (neuropsin/ovasin) gene: identification of two novel splice variants and its prognostic value in ovarian cancer Clin Cancer Res 7 806811 11309326

[bib36] MaguireTMSheringSGDugganCMMcDermottEWO'HigginsNJDuffyMJ1998 High levels of cathepsin B predict poor outcome in patients with breast cancer Int J Biol Markers 13 139144 10079387 10.1177/172460089801300303

[bib37] MantelN1966 Evaluation of survival data and two new rank order statistics arising in its consideration Cancer Chemother Rep 50 163170 5910392

[bib38] ObiezuCVScorilasAKatsarosDMassobrioMYousefGMFracchioliSRigault de la LongraisIAArisioRDiamandisEP2001 Higher human kallikrein gene 4 (KLK4) expression indicates poor prognosis of ovarian cancer patients Clin Cancer Res 7 23802386 11489816

[bib39] ParkinDMPisaniPFerlayJ1999 Estimates of the worldwide incidence of 25 major cancers in 1990 Int J Cancer 80 827841 10074914 10.1002/(sici)1097-0215(19990315)80:6<827::aid-ijc6>3.0.co;2-p

[bib40] PetoRBorehamJClarkeMDaviesCBeralV2000 UK and USA breast cancer deaths down 25% in year 2000 at ages 20–69 years Lancet 355 1822 10.1016/S0140-6736(00)02277-7 10832853

[bib41] RittenhouseHGFinlayJAMikolajczykSDPartinAW1998 Human Kallikrein 2 (hK2) and prostate-specific antigen (PSA): two closely related, but distinct, kallikreins in the prostate Crit Rev Clin Lab Sci 35 275368 9759557 10.1080/10408369891234219

[bib42] RossJSFletcherJA1998 The HER-2/neu oncogene in breast cancer: Prognostic factor, predictive Factor, and target for therapy Oncologist 3 237252 10388110

[bib43] RussoIHRussoJ1998 Role of hormones in mammary cancer initiation and progression J Mammary Gland Biol Neoplasia 3 4961 10819504 10.1023/a:1018770218022

[bib44] ScorilasAYotisJPaterasCTrangasTTalieriM1999 Predictive value of c-erbB-2 and cathepsin-D for Greek breast cancer patients using univariate and multivariate analysis Clin Cancer Res 5 815821 10213217

[bib45] SmithRAVon EschenbachACWenderALevinBByersTRothenbergerDBrooksDCreasmanWCohenCRunowiczCSaslowDCokkinidesVEyreH2001 American Cancer Society guidelines for early detection of cancer: update of early detection guidelines for prostate, colorectal, and endometrial cancers, and update 2001: testing for early lung cancer detection CA Cancer J Clin 51 738 10.3322/canjclin.51.1.38 11577479

[bib46] SoreideJALeaOAVarhaugJESkarsteinAKvinnslandS1992 Androgen receptors in operable breast cancer: relation to other steroid hormone receptors, correlations to prognostic factors and predictive value for effect of adjuvant tamoxifen treatment Eur J Surg Oncol 18 112118 1582503

[bib47] XieBTsaoSWWongYC1999 Sex hormone-induced mammary carcinogenesis in female noble rats: the role of androgens Carcinogenesis 20 15971606 10426813 10.1093/carcin/20.8.1597

[bib48] YousefGMChangADiamandisEP2000a Identification and characterization of KLK-L4, a new kallikrein-like gene that appears to be down-regulated in breast cancer tissues J Biol Chem 275 1189111898 10766816 10.1074/jbc.275.16.11891

[bib49] YousefGMChangAScorilasADiamandisEP2000b Genomic organization of the human kallikrein gene family on chromosome 19q13.3-q13.4 Biochem Biophys Res Commun 276 125133 11006094 10.1006/bbrc.2000.3448

[bib50] YousefGMMagklaraADiamandisEP2000c KLK12 is a novel serine protease and a new member of the human kallikrein gene family – differential expression in breast cancer Genomics 69 331341 11056051 10.1006/geno.2000.6346

[bib51] YousefGMDiamandisEP2001 The new human tissue kallikrein gene family: structure, function, and association to disease Endocr Rev 22 184204 11294823 10.1210/edrv.22.2.0424

[bib52] YousefGMKyriakopoulouLGScorilasAFracchioliSGhiringhelloBZarghooniMChangADiamandisMGiardinaGHartwickWJRichiardiGMassobrioMDiamandisEPKatsarosD2001a Quantitative expression of the human kallikrein gene 9 (KLK9) in ovarian cancer: a new independent and favorable prognostic marker Cancer Res 61 78117818 11691797

[bib53] YousefGMMagklaraAChangAJungKKatsarosDDiamandisEP2001b Cloning of a new member of the human kallikrein gene family, KLK14, which is down-regulated in different malignancies Cancer Res 61 34253431 11309303

[bib54] YousefGMScorilasAJungKAshworthLKDiamandisEP2001c Molecular cloning of the human kallikrein 15 gene (KLK15). Up-regulation in prostate cancer J Biol Chem 276 5361 11010966 10.1074/jbc.M005432200

[bib55] YousefGMDiamandisEP2002 Human kallikreins: common structural features, sequence analysis and evolution Curr GenomIn Press

[bib56] YuHGiaiMDiamandisEPKatsarosDSutherlandDJLevesqueMARoagnaRPonzoneRSismondiP1995 Prostate-specific antigen is a new favorable prognostic indicator for women with breast cancer Cancer Res 55 21042110 7538047

